# Semaglutide or Tirzepatide and Optic Nerve and Visual Pathway Disorders in Type 2 Diabetes

**DOI:** 10.1001/jamanetworkopen.2025.26327

**Published:** 2025-08-11

**Authors:** Lindsey Wang, Nora D. Volkow, David C. Kaelber, Rong Xu

**Affiliations:** 1 Center for Science, Health, and Society, Case Western Reserve University School of Medicine, Cleveland, Ohio; 2 National Institute on Alcohol Abuse and Alcoholism, National Institutes of Health, North Bethesda, Maryland; 3 Center for Clinical Informatics Research and Education, MetroHealth System, Cleveland, Ohio; 4 Center for Artificial Intelligence in Drug Discovery, Case Western Reserve University School of Medicine, Cleveland, Ohio

## Abstract

**Importance:**

Semaglutide and tirzepatide, the new generation of glucagon-like peptide-1 receptor agonist used to treat type 2 diabetes and obesity, were recently reported to be associated with increased risk of nonarteritic anterior ischemic optic neuropathy (NAION). However, reported associations were inconsistent, and it remains unknown if semaglutide or tirzepatide is associated with other disorders of the optic nerve and visual pathways.

**Objective:**

To examine associations of treatment with either semaglutide or tirzepatide with first-time diagnoses of optic nerve and visual pathway disorders in patients with type 2 diabetes.

**Design, Setting, and Participants:**

This cohort study emulated target trials based on a nationwide, population-based database of US patient electronic health records from December 2017 to January 2023. Models were adjusted by propensity score matching. Eligible patients had type 2 diabetes and no prior diagnosis of eye disorders and were prescribed semaglutide, tirzepatide, or other antidiabetic medications.

**Exposures:**

Prescription of semaglutide or tirzepatide vs other antidiabetic medications (insulins, metformin, dipeptidyl-peptidase-4 inhibitors, sodium-glucose cotransporter-2 inhibitors, sulfonylureas, thiazolidinediones, and other glucagon-like peptide 1 receptor agonists).

**Main Outcomes and Measures:**

First-time diagnoses of NAION and other optic nerve disorders during a 2-year follow-up were examined using Cox proportional hazards and Kaplan-Meier survival analyses.

**Results:**

After propensity score matching among 1 511 637 eligible patients, the study population comprised 159 398 matched patients with type 2 diabetes (mean [SD] age, 56.5 [13.3] years; 83 123 women [52.15%]), including 79 699 patients in the semaglutide or tirzepatide group and 79 699 patients in the comparison group. During the 2-year follow-up, there were 35 patients (0.04%) with NAION in the semaglutide or tirzepatide group and 19 patients (0.02%) with NAION in the matched comparison group (hazard ratio, 1.76 [95% CI, 1.01-3.07]). There were 93 patients (0.12%) with other optic nerve disorders in the semaglutide or tirzepatide group and 54 patients (0.07%) with other optic nerve disorders in the matched comparison group (hazard ratio, 1.65 [95% CI, 1.18-2.31]). No association was found with other disorders of the optic nerve or visual pathways.

**Conclusions and Relevance:**

In this study of patients with type 2 diabetes who had no prior eye disorders, patients prescribed semaglutide or tirzepatide had an increased risk of NAION and other optic nerve disorders, although the overall risk was low. These findings highlight the need for close monitoring of these conditions.

## Key Points

**Question:**

Is treatment with either semaglutide or tirzepatide associated with an increased risk of nonarteritic anterior ischemic optic neuropathy and other optic nerve and visual pathway disorders?

**Findings:**

This cohort study emulated target trials among 1 511 637 eligible patients with type 2 diabetes by comparing treatment with either semaglutide or tirzepatide vs other antidiabetic medications. Semaglutide or tirzepatide was associated with increased risk of nonarteritic anterior ischemic optic neuropathy and other optic nerve disorders, but the overall risk was low.

**Meaning:**

This analysis of patient electronic health record data suggests an increased risk of optic nerve disorders associated with semaglutide or tirzepatide in patients with type 2 diabetes, highlighting the need for close monitoring of these conditions.

## Teaser

This cohort study investigates the association of semaglutide and tirzepatide with nonarteritic anterior ischemic optic neuropathy and other optic nerve and visual pathway disorders in patients with type 2 diabetes.

## Introduction

Glucagon-like peptide-1 receptor agonist (GLP-1RA) medications have transformed the treatment of type 2 diabetes and obesity, with associated reductions in cardiovascular and nephrological complications.^1,2^ Semaglutide and tirzepatide are second-generation GLP-1RA medications approved by the Food and Drug Administration for the treatment of diabetes and obesity.^3,4,5,6^ The main associated adverse outcomes are gastrointestinal outcomes, including nausea, vomiting, diarrhea, gastroparesis, and constipation. Studies also reported potential associations of GLP-1RAs with thyroid C-cell tumors and pancreatitis.^7,8^

Nonarteritic anterior ischemic optic neuropathy (NAION) is characterized by loss of vision caused by loss of blood flow to the optic nerve.^9^ Its prevalence is higher in patients with diabetes, obesity, hypertension, hyperlipidemia, cerebrovascular disease, and obstructive sleep apnea.^10^ Although the etiopathogenesis of NAION is not fully understood, decreased arterial perfusion of the optic nerve and the associated swelling and compression of the optic nerve are implicated.^9^ Several retrospective studies from 2024 and 2025 reported a potential association of semaglutide with NAION in patients with diabetes and patients with obesity.^11,12,13,14,15^ However, a meta-analysis of randomized clinical trials^16^ did not detect an association of GLP1-RA therapy, including semaglutide, with NAION. A rare condition, NAION has an incidence rate of 2.5 to 11.8 incidents per 100 000 individuals.^9^ These inconsistent findings may be due to limited sample sizes and different study designs. Importantly, it remains unknown if semaglutide or tirzepatide is associated with other optic nerve and visual pathway disorders. This study leveraged a nationwide, multicenter database of electronic health records (EHRs) of more than 118 million US patients to conduct rigorous target trial emulation in patients with type 2 diabetes to examine associations of semaglutide or tirzepatide with optic nerve and visual pathway disorders, including NAION.

## Methods

This retrospective cohort study was exempt from ethical review and informed consent because it was a secondary analysis of existing data, did not involve intervention or interaction with human participants, and used deidentified data per the deidentification standard defined in section §164.514(a) of the HIPAA Privacy Rule. The process by which the data were deidentified is attested to through a formal determination by a qualified expert as defined in section §164.514(b)(1) of the HIPAA Privacy Rule. This study followed the Strengthening the Reporting of Observational Studies in Epidemiology (STROBE) reporting guideline.

### Specification of Target Trials

#### Study Overview

We compared semaglutide or tirzepatide with other antidiabetic medications for the associated risk of optic nerve and visual pathway disorders in patients with type 2 diabetes who had no prior diagnosis of any eye disorder (determined by the absence of *International Statistical Classification of Diseases and Related Health Problems, Tenth Revision *[*ICD-10*] codes H00-H59, “Diseases of the eye and adnexa,” documented in EHRs) using a target trial emulation framework.^17,18^ Other antidiabetic medications included insulin, metformin, dipeptidyl-peptidase-4 inhibitors, sodium-glucose cotransporter-2 inhibitors, sulfonylureas, thiazolidinediones, and other GLP-1RAs (albiglutide, dulaglutide, exenatide, liraglutide, and lixisenatide). Key protocol components are listed in eTable 1 in Supplement 1.

Semaglutide and tirzepatide are newer and more potent GLP-RAs to treat type 2 diabetes and obesity.^19^ To examine differences between semaglutide or tirzepatide and first-generation GLP-1RAs in their associated risk for NAION and other optic nerve and visual pathway disorders, we performed a head-to-head comparison of semaglutide or tirzepatide with other GLP-1RAs and a head-to-head comparison of semaglutide with tirzepatide.

#### Eligibility Criteria

Eligibility criteria for all target trials included a prior diagnosis of type 2 diabetes, a recent medical encounter for type 2 diabetes diagnosis in the past year (*active type 2 diabetes*), prescriptions of antidiabetic medications (semaglutide, tirzepatide, or other antidiabetic medications) between December 2017 and January 2023, and diagnosis with at least 1 condition among the prescription guidelines for semaglutide and tirzepatide (eg, obesity, hypertension, hypercholesterolemia, heart disease, stroke, kidney disease, or an A_1C_ level ≥8.5%).^20,21^ Exclusion criteria included a prior diagnosis of any eye disorder, including optic nerve and visual pathway disorders; initiation of semaglutide or tirzepatide and comparison antidiabetic medications at the same time; and certain medical conditions (ie, pancreatitis, type 1 diabetes, thyroid cancer, and gastroparesis) based on contraindications, warnings, and limited use information for semaglutide and tirzepatide (eTable 2 in Supplement 1).^20,21^

### Treatment Strategies

In each target trial, treatment strategies were the initiation of semaglutide or tirzepatide use at baseline (time zero, index event, or time of randomization) or the initiation of comparison antidiabetic medication use at baseline but not both strategies. The treatment strategy was assigned at baseline, regardless of medication use adherence, medication switch, or add-on.

### Study Outcomes

Main outcomes were the first-time diagnosis of overall and subcategories of disorders of optic nerve and visual pathways: (1) disorders of optic nerve and visual pathways (*ICD-10* code H46-H47), (2) optic neuritis (H46), (3) other disorders of optic nerve and visual pathways (H47), (4) disorders of optic nerve (H47.0), (5) NAION (H47.01), (6) other optic nerve disorders (H47.02, H47.03, H47.09), (7) papilledema (H47.1), (8) optic atrophy (H47.2), and (9) other disorders of optic disc (H47.3). Each outcome was analyzed separately, with no multiple comparisons or competing outcomes. For each outcome, eligible patients were followed up starting after the index event until the occurrence of the outcome, death, loss to follow-up, or 2 years after the index event, whichever occurred first. Details of diagnosis codes for study outcomes are in eTable 3 in Supplement 1.

### Statistical Analysis

The estimated outcomes of interest represent the association of treatment strategy assignments with outcomes in the intention-to-treat population. Cumulative incidences were estimated using Kaplan-Meier survival analysis. Cox proportional hazard analyses were used to compare rates of time to events daily during follow-up after the index event. Censoring was applied in Kaplan-Meier survival and Cox proportional hazard analyses. Hazard ratios (HRs) and 95% CIs were calculated; HRs were considered statistically significant at the *P* < .05 level if the 95% CIs did not include 1.

We used TriNetX, a global federated health research network providing access to statistics on EHRs (diagnoses, procedures, medications, laboratory values, and genomic information) from approximately 118 million patients in 69 large health care organizations covering diverse geographic regions, age, race and ethnicity groups, income and insurance groups, and clinical settings.^22^

We explicitly emulated target trials described previously using data and built-in analytic functions on the TriNetX Analytics platform. We previously performed emulation target trials and cohort studies using the TriNetX platform to examine the association of GLP-1RAs, including semaglutide, with Alzheimer disease,^23^ substance use disorders,^24,25,26,27^ suicidal ideation,^28^ and cancers.^29,30,31,32^

Available data elements of EHRs included extensive information on demographics, diagnoses, medications, procedures, laboratory tests, visits, and socioeconomic and lifestyle information. All covariates were binary, categorical, or continuous but were essentially guaranteed to exist (more details on TriNetX are in the eAppendix in Supplement 1). Sex, race, and ethnicity data were self-reported from contributing health care systems. Race and ethnicity data were mapped according to Office of Management and Budget standards into race (American Indian or Alaska Native, Asian, Black or African American, Native Hawaiian or Other Pacific Islander, White, and unknown race) and ethnicity (Hispanic or Latinx, not Hispanic or Latinx, or unknown ethnicity). Missing sex values are represented using *unknown sex*. Missing data for race and ethnicity are presented as *unknown race* or *unknown ethnicity*. Race and ethnicity as social constructs were assessed due to their association with health disparities and the differential impact of structural disadvantages.

Each component of the target trial was emulated using EHRs from the TriNetX Analytics platform (more details of target trial emulation components are in eTables 1-4 and eFigure 1 in Supplement 1). Patients were classified into drug treatment groups (semaglutide or tirzepatide vs other antidiabetic medications, including insulin, metformin, dipeptidyl-peptidase-4 inhibitors, sodium-glucose cotransporter-2 inhibitors, sulfonylureas, thiazolidinediones, and other GLP-1RAs) based on the first prescription in the study period (December 2017 to January 2023), which was the baseline, time zero, or index event. Eligibility criteria and more than 50 baseline covariates were evaluated at baseline based on their status on or before time zero. Baseline covariates included demographics, adverse determinants of socioeconomic health, lifestyle factors, medical conditions and medications that are risk factors associated with NAION and optic nerve and visual pathway disorders,^33,34^ prior prescriptions of antidiabetic medications, and medical encounters. For each comparison, exposure and comparison groups were propensity score matched for covariates at the baseline to emulate randomization. We used the TriNetX Analytics built-in 1:1 propensity score–matching function, which uses nearest neighbor greedy matching with a caliper of 0.1 times the SD for baseline covariates.^17^ This means that for 2 patients to match, the difference between their propensity scores must be no greater than a tolerance level based on a predefined caliper of 0.1 times the pooled SDs of the propensity scores. For each variable, standardized mean difference was calculated. A threshold of a standardized mean difference less than 0.1 was used to indicate balanced variables.

#### Sensitivity Analyses

Several negative control outcomes were used for quantifying potential residual bias. They were first diagnosis of congenital eye diseases (Q10-Q15), first-time diagnosis of any eye diseases (H00-H59), outpatient medical encounters, and overall medical encounters. Congenital eye diseases can be caused by genetic mutations, exposure to drugs or alcohol during pregnancy, or maternal infections, and our study populations comprised mostly adult patients with type 2 diabetes (mean age, 56.5 years); therefore, we assumed that semaglutide or tirzepatide was not associated with an altered risk of congenital eye diseases. However, whether semaglutide or tirzepatide was associated with an altered risk of overall eye diseases remained unknown. Outpatient and overall medical encounters were used to measure overall health care use. As part of sensitivity analyses, we also calculated the *E*-value, which measured how susceptible the study results were to unmeasured or uncontrolled confounding.^18^ The *E*-value for the observed outcome and CIs using HR and 95% CI was calculated with the following equation:

*E*-value (outcome) = HR + √(HR × [HR − 1])

*E*-value (lower CI, or LL) = LL + √(LL × [LL − 1])

*E*-value (upper CI, or UL) = UL + √(UL × (UL-1)).

#### Analytics Platform and Statistical Software

Data were collected and analyzed in February 2025 within the TriNetX Analytics platform. All statistical analyses, including propensity score matching, Kaplan-Meier survival analysis, and Cox proportional hazards analyses, were done using built-in functions within the TriNetX Analytics platform that are implemented using the R Survival package^35^ version 3.2-3in R statistical software version 4.0.2 (R Project for Statistical Computing) and libraries and utilities for in Python programming language version 3.7 (Python Software Foundation) and Java programming language version 11.0.16 (Oracle Corporation).

## Results

### Study Populations

**Figure 1.**
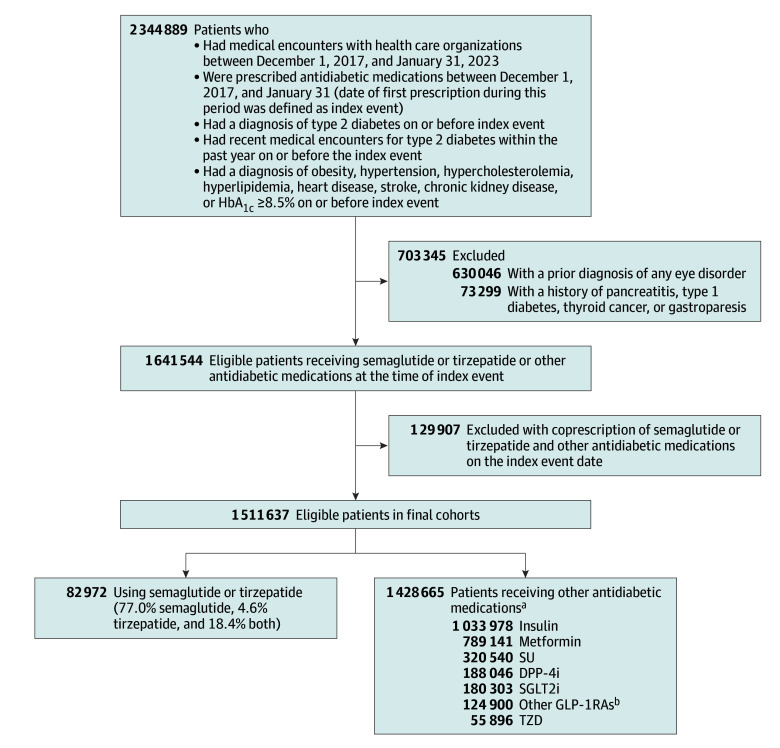
Study Flowchart DPP-4i indicates dipeptidyl-peptidase-4 inhibitor; GLP-1RA, glucagon-like peptide-1 receptor agonist; SGLT2i, sodium-glucose cotransporter-2 inhibitor; SU, sulfonylurea; TZD, thiazolidinedione. ^a^The combined total of 1 428 665 patients in the comparison arm is not a sum of patients from each antidiabetic medication group because a patient could be prescribed more than 1 antidiabetic medication during the study period. However, there was no overlap between the semaglutide or tirzepatide group and comparison medication groups. ^b^Other GLP-1RAs included albiglutide (1108 patients [0.89%]), dulaglutide (83 501 patients [66.85%]), exenatide (17 046 patients [13.65%]), liraglutide (53 189 patients [42.59%]), and lixisenatide (2465 patients [1.97%]).

Figure 1 is the flowchart showing cohort construction. The study included 1 511 637 eligible patients with type 2 diabetes without prior diagnosis of any eye disorder who were prescribed antidiabetic medications, including 82 972 patients prescribed semaglutide or tirzepatide (63 926 patients [77.05%] prescribed semaglutide, 3815 patients [4.60%] prescribed tirzepatide, and 15 231 patients [18.36%] prescribed both medications) and 1 428 665 patients prescribed other antidiabetic medications. Before propensity score matching, semaglutide or tirzepatide and comparison groups differed by demographics, comorbidities, and medications. After propensity score matching, the study population comprised 159 398 matched patients with type 2 diabetes (mean [SD] age, 56.5 [13.3] years; 83 123 women [52.15%]; 11 487 Hispanic [7.21%]; 7157 Asian [4.49%], 25 121 Black [15.76%], and 102 381 White [64.23%]), including 79 699 patients in the semaglutide or tirzepatide group and 79 699 patients in the comparison group. Matched groups were balanced (Table; eFigures 2-3 in Supplement 1) and had high comorbidities, including 92 232 patients with obesity (57.80%) and 121 317 patients with hypertension (76.11%).

**Table.** Patient Characteristics

Characteristic	Patients, No. (%)^a^					
	Before propensity score matching (N = 1 511 637)			After propensity score matching (N = 159 398)		
	Semaglutide or tirzepatide (n = 82 972)	Other antidiabetic medications (n = 1 428 665)	SMD	Semaglutide or tirzepatide (n = 79 699)	Other antidiabetic medications (n = 79 699)	SMD
Age at index event, mean (SD), y	56.6 (12.4)	63.3 (14.0)	0.51^b^	56.8 (12.4)	56.2 (14.1)	0.04
Sex						
Female	42 994 (51.8)	636 197 (44.5)	0.15^b^	41 210 (51.7)	41 913 (52.6)	0.02
Male	35 017 (42.2)	738 209 (51.7)	0.19^b^	33 813 (42.4)	33 421 (41.9)	0.01
Unknown	4958 (6.0)	54 288 (3.8)	0.10^b^	4676 (5.9)	4365 (5.5)	0.02
Ethnicity						
Hispanic or Latinx	5849 (7.0)	134 959 (9.4)	0.09	5679 (7.1)	5808 (7.3)	0.006
Not Hispanic or Latinx	58 747 (70.8)	915 044 (64.0)	0.15^b^	56 406 (70.8)	57 119 (71.7)	0.02
Unknown	18 375 (22.1)	378 691 (26.5)	0.10^b^	17 614 (22.1)	16 772 (20.8)	0.03
Race						
Asian	3983 (4.7)	79 663 (5.6)	0.04	3725 (4.7)	3432 (4.3)	0.02
Black	12 904 (15.6)	257 078 (18.0)	0.07	12 506 (15.7)	12 615 (15.8)	0.004
White	52 963 (63.8)	851 259 (59.6)	0.09	50 829 (63.8)	51 552 (64.7)	0.02
Unknown	8563 (10.3)	158 577 (11.1)	0.03	8189 (10.3)	7830 (9.8)	0.02
Adverse socioeconomic determinants of health^c^	2460 (3.0)	26 741 (1.9)	0.07	2305 (2.9)	2321 (2.9)	0.001
Problems related to lifestyle^d^	5941 (7.2)	62 518 (4.4)	0.12^b^	5645 (7.1)	5615 (7.0)	0.001
Type 2 diabetes complications						
Kidney	12 497 (15.1)	178 331 (12.5)	0.08	11 839 (14.9)	11 609 (14.6)	0.008
Ophthalmic	2993 (3.6)	28 221 (2.0)	0.10^b^	2834 (3.6)	2919 (3.7)	0.006
Neurological	13 277 (16.0)	143 041 (10.0)	0.18^b^	12 597 (15.8)	12 929 (16.2)	0.01
Circulatory	6337 (7.6)	67 035 (4.7)	0.12^b^	5933 (7.4)	5839 (7.3)	0.005
Other specified	40 942 (49.3)	350 065 (24.5)	0.53^b^	38 446 (48.2)	38 877 (48.8)	0.01
Unspecified	9368 (11.3)	82 479 (5.8)	0.20^b^	8830 (11.1)	9001 (11.3)	0.007
Preexisting medical conditions						
Overweight or obesity	48 286 (58.2)	353 004 (24.7)	0.72^b^	45 550 (57.2)	46 682 (58.6)	0.03
Metabolic disorder	65 428 (78.9)	798 209 (55.9)	0.51^b^	62 445 (78.4)	63 538 (79.7)	0.03
Hypertension	62 888 (75.8)	868 192 (60.8)	0.33^b^	60 213 (75.6)	61 104 (76.7)	0.03
Hypotension	3444 (4.2)	68 372 (4.8)	0.03	3336 (4.2)	3284 (4.1)	0.003
Disorder of lipoprotein metabolism or other lipidemia	62 182 (74.9)	703 727 (49.3)	0.55^b^	59 250 (74.3)	60 506 (75.9)	0.04
Ischemic heart disease	15 120 (18.2)	296 180 (20.7)	0.06	14 632 (18.4)	14 346 (18.0)	0.009
Other form of heart disease	21 779 (26.2)	390 040 (27.3)	0.02	20 984 (26.3)	20 465 (25.7)	0.02
Cerebrovascular disease	5583 (6.7)	135 799 (9.5)	0.10^b^	5418 (6.8)	5285 (6.6)	0.007
Kidney disease	13 680 (16.5)	277 510 (19.4)	0.08	13 222 (16.6)	12 919 (16.2)	0.01
Atherosclerosis	3491 (4.2)	63 244 (4.4)	0.01	3366 (4.2)	3364 (4.2)	<.001
Sleep apnea	24 168 (29.1)	170 150 (11.9)	0.44^b^	22 660 (28.4)	23 114 (29.0)	0.01
Migraine	5853 (7.1)	32 631 (2.3)	0.23^b^	5411 (6.8)	5443 (6.8)	0.002
Coagulation defect, purpura, or other hemorrhagic condition	3934 (4.7)	70 392 (4.9)	0.009	3790 (4.8)	3715 (4.7)	0.004
Alcohol use disorder	1728 (2.1)	40 670 (2.8)	0.05	1689 (2.1)	1657 (2.1)	0.003
Tobacco use disorder	10 215 (12.3)	162 128 (11.3)	0.03	9853 (12.4)	9819 (12.3)	0.001
Prior medications						
Antihypertensive	12 413 (15.0)	172 321 (12.1)	0.09	11 907 (14.9)	11 812 (14.8)	0.003
Antihypertensive, other	17 331 (20.9)	197 027 (13.8)	0.19^b^	16 482 (20.7)	16 456 (20.6)	0.001
Antiarrhythmic	37 024 (44.6)	399 862 (28.0)	0.35^b^	35 081 (44.0)	34 735 (43.6)	0.009
Amphetamine	2441 (2.9)	7785 (0.5)	0.18^b^	2169 (2.7)	2093 (2.6)	0.006
Aspirin	25 052 (30.2)	316 568 (22.2)	0.18^b^	24 024 (30.1)	24 164 (30.3)	0.004
Sildenafil	5157 (6.2)	30 323 (2.1)	0.21^b^	4801 (6.0)	4802 (6.0)	<.001
Tadalafil	3204 (3.9)	17 719 (1.2)	0.17^b^	2985 (3.7)	3021 (3.8)	0.002
Amiodarone	1685 (2.0)	26 057 (1.8)	0.02	1629 (2.0)	1604 (2.0)	0.002
Interferon	77 (0.1)	1222 (0.1)	0.002	72 (0.1)	116 (0.1)	0.02
Insulin	34 773 (41.9)	251 055 (17.6)	0.55^b^	32 650 (41.0)	33 969 (42.6)	0.03
Metformin	57 029 (69.0)	295 891 (20.7)	1.11^b^	54 032 (67.8)	56 665 (71.1)	0.07
DPP-4 inhibitor	15 991 (19.3)	73 846 (5.2)	0.44^b^	14 865 (18.7)	15 281 (19.2)	0.01
SGLT2 inhibitor	21 453 (25.9)	24 567 (1.7)	0.75^b^	18 578 (23.3)	15 947 (20.0)	0.08
Sulfonylurea	23 155 (27.9)	141 814 (9.9)	0.47^b^	21 741 (27.3)	22 623 (28.4)	0.03
Other GLP-1RA	20 960 (25.3)	28 164 (2.0)	0.72^b^	18 289 (23.3)	16 112 (20.0)	0.08
Medical encounters						
Outpatient medical visit	69 369 (83.6)	918 705 (64.3)	0.45^b^	66 567 (83.5)	69 213 (86.8)	0.09
Inpatient medical visit	22 515 (27.1)	431 388 (30.2)	0.07	21 785 (27.3)	21 835 (27.4)	0.001
Emergency visit	31 489 (38.0)	456 293 (31.9)	0.13^b^	30 057 (37.7)	29 617 (37.2)	0.01

Abbreviations: DPP-4, dipeptidyl peptidase 4; GLP-1RA, glucagon-like peptide 1 agonist; SGLT2, sodium-glucose cotransporter 2; SMD, standardized mean difference.

^a^ Shown are groups before and after propensity score matching for the listed variables. The status of variables was based on the presence of related clinical codes any time up to 1 day before the index event (first prescription of semaglutide or insulin during December 2017 to January 2023).

^b^ SMD greater than 0.1, a threshold indicating cohort imbalance.

^c^ Adverse socioeconomic determinants of health included housing and economic circumstances, upbringing, education, physical environment, and social environment.

^d^ Problems with lifestyle included tobacco use, lack of physical exercise, inappropriate diet and eating habits, and others.

### Associations of Semaglutide or Tirzepatide With Optic Nerve and Visual Pathway Disorders

**Figure 2.**
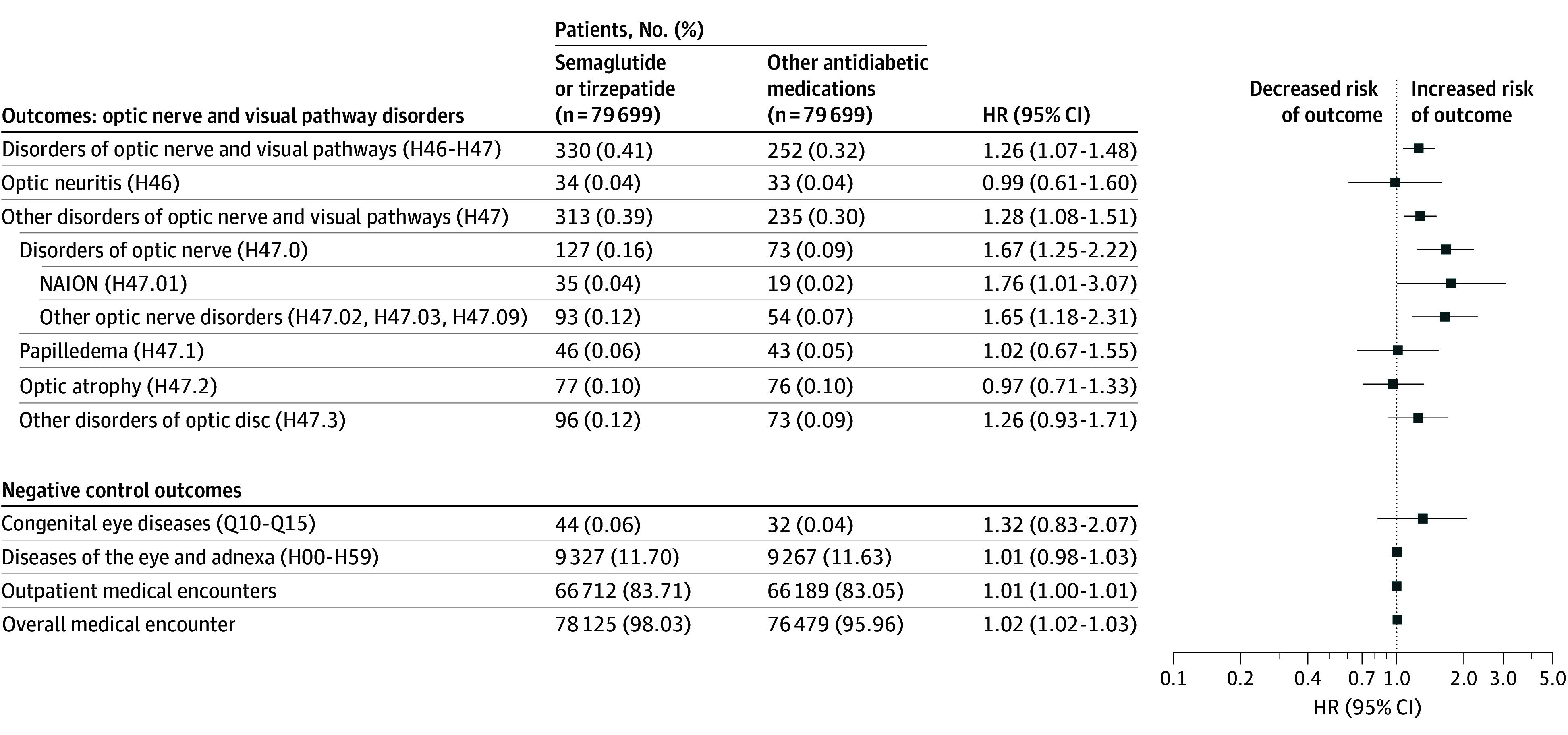
Risk of Optic Nerve and Visual Pathway Disorders for Semaglutide or Tirzepatide vs Other Antidiabetic Medications Exposure and comparison groups were propensity score matched for variables listed in Table 1, and the status of variables was based on the presence of related clinical codes any time up to 1 day before the index event (first prescription of semaglutide or tirzepatide vs comparison medication classes during December 2017 to January 2023). Individuals in matched groups were followed up after the index event until the occurrence of the outcome, death, loss to follow-up, or 2 years after the index event, whichever occurred first. Hazard rates were calculated using a Cox proportional hazards model with censoring applied. Overall risk was the number of patients with outcomes during the follow-up time window divided by the number of patients in the cohort at the beginning of the time window. HR indicates hazard ratio; NAION, nonarteritic anterior ischemic optic neuropathy.

During the 2-year follow-up, there were 35 patients (0.04%) with NAION in the semaglutide or tirzepatide group and 19 patients with NAION (0.02%) in the matched comparison group (HR, 1.76 [95% CI, 1.01-3.07]). There were 93 patients (0.12%) with other optic nerve disorders (H47.02, H47.03, and H47.09) in the semaglutide or tirzepatide group and 54 patients (0.07%) with these disorders in the matched comparison group (HR, 1.65 [95% CI, 1.18-2.31]). There was no association of semaglutide or tirzepatide with optic neuritis, papilledema, or optic atrophy. Patients in the semaglutide or tirzepatide group had an increased incidence of other optic disc disorders, but this difference was not statistically significant (Figure 2).

For sensitivity analysis, semaglutide or tirzepatide was not associated with any of 4 negative control outcomes, including medical encounters for congenital eye diseases diagnosis, medical encounters for any eye disease diagnosis, outpatient medical encounters, and all medical encounters (Figure 2). The *E*-value was 2.92 (95% CI, 1.11-5.59) for NAION, greater than the previously reported HRs of 1.65 for hypertension and 2.46 for hypercoagulable states, 2 major risk factors associated with NAION.^36^ However, reported associations of risk factors with NAION varied among studies.^33,37^ The *E*-value was 2.69 (95% CI, 1.64-4.05) for other optic nerve disorders.

**Figure 3.**
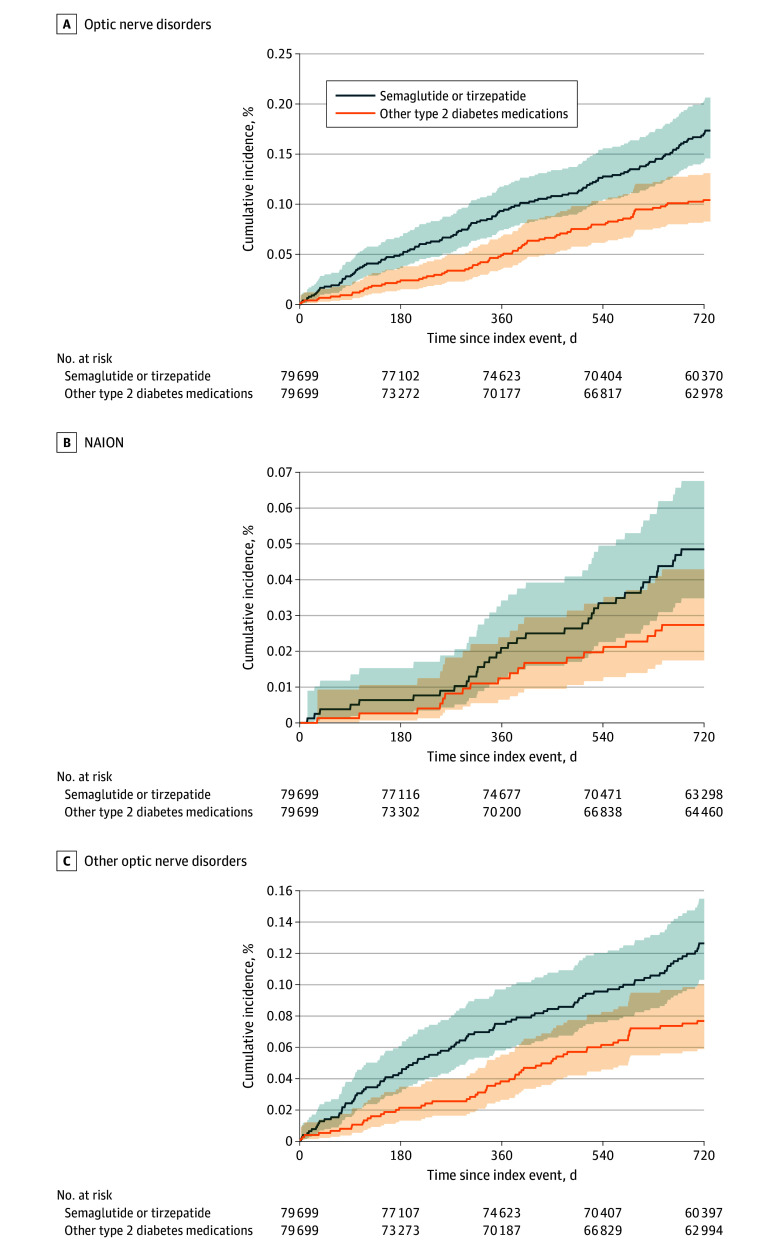
Cumulative Incidence of Optic Nerve and Visual Pathway Disorders The 2-year cumulative incidence curves are shown for A, optic nerve disorders, B, nonarteritic anterior ischemic optic neuropathy (NAION), and C, other optic nerve disorders for the propensity score matched semaglutide or tirzepatide group and other antidiabetic medication groups in patients with type 2 diabetes who had no prior diagnosis of eye disorders.

The 2-year cumulative incidence curves of optic nerve disorders, NAION, and other optic nerve disorders are shown in Figure 3. For the outcome of optic nerve disorders, the mean (SD) follow-up time was 671.3 (50.9) days for the semaglutide or tirzepatide group and 643.0 (77.3) days for the comparison group. For the outcome of NAION, the mean (SD) follow-up time was 674.8 (49.2) days for the semaglutide or tirzepatide group and 647.5 (74.3) days for the comparison group. For the outcome of other optic nerve disorders, the mean (SD) follow-up time was 671.9 (50.5) days for the semaglutide or tirzepatide group and 643.8 (76.6) days for the comparison group. The slope for the semaglutide or tirzepatide group was consistently higher than that for the comparison group during the 2-year follow-up, indicating a sustained association of semaglutide or tirzepatide with the risk of developing optic nerve disorders.

### Head-to-Head Comparisons With Other GLP-1RAs and Semaglutide vs Tirzepatide

**Figure 4.**
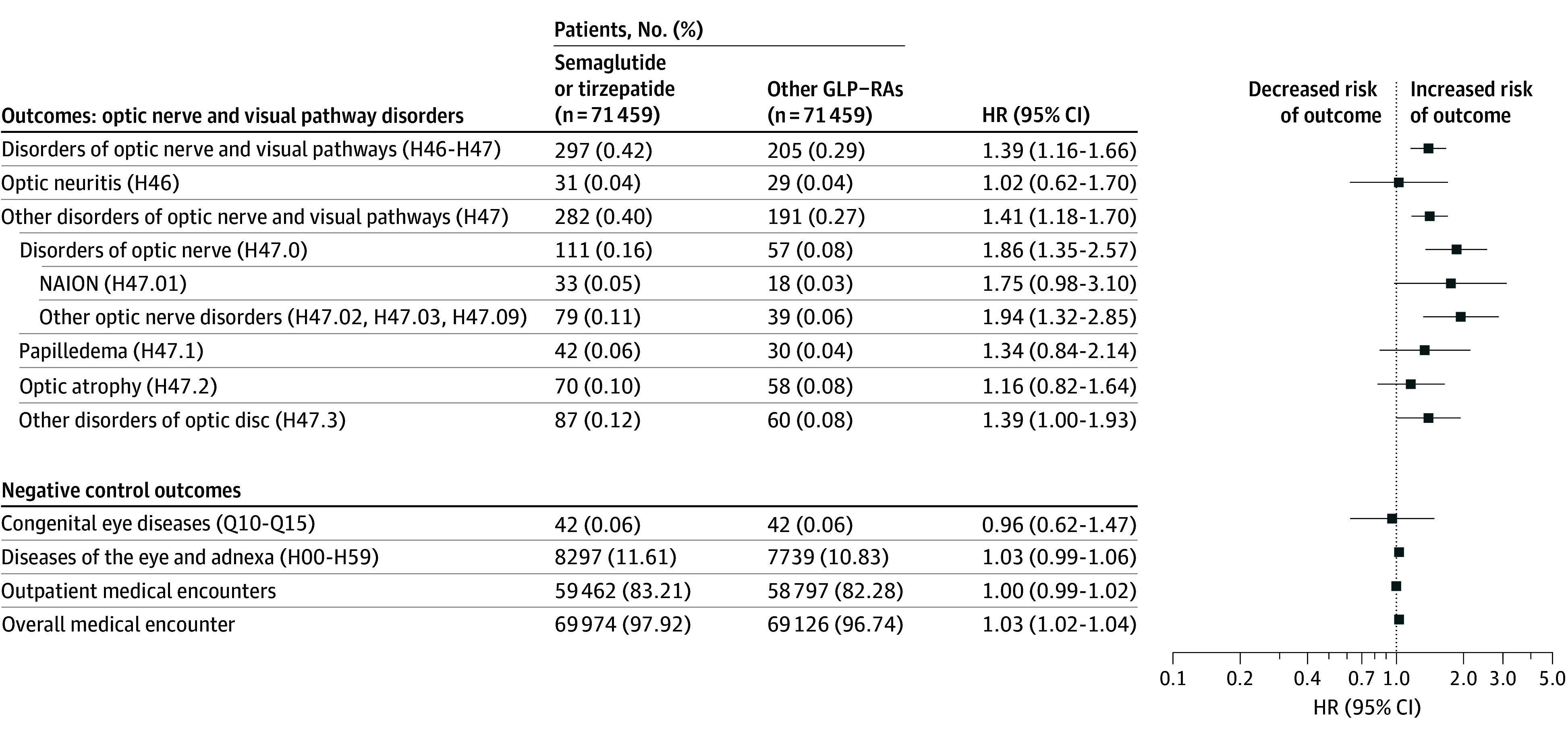
Risk of Optic Nerve and Visual Pathway Disorders for Semaglutide or Tirzepatide vs Other Glucagon-Like Peptide 1 Agonists (GLP-1RAs) Exposure and comparison groups were propensity score matched. HR indicates hazard ratio; NAION, nonarteritic anterior ischemic optic neuropathy.

To examine potential differential associations of new and older-generation GLP-1RAs with optic nerve and visual pathway disorders, we performed head-to-head comparison of semaglutide or tirzepatide with other GLP-1RAs (albiglutide, dulaglutide, exenatide, liraglutide, and lixisenatide). Before propensity score matching, the semaglutide or tirzepatide cohort and other GLP-1RA cohorts included 82 972 and 124 908 patients, respectively. The cohorts differed in demographics, comorbidities, and medications. After matching, each cohort included 71 459 patients and the 2 cohorts were balanced (eTable 5 in Supplement 1). Compared with other GLP-1RAs, treatment with semaglutide or tirzepatide was not associated with risk of NAION (HR, 1.75 [95% CI, 0.98-3.10]) but was associated with increased risk of other optic nerve disorders (HR, 1.94 [95% CI, 1.32-2.85]) and other optical disc orders (HR, 1.39 [95% CI, 1.00-1.93]) (Figure 4).

Semaglutide was then directly compared with tirzepatide. After matching, there were 3806 patients in each cohort. There were 6 patients with optic nerve disorders in the semaglutide cohort, including 2 patients with NAION, and 4 patients with optic nerve disorders in the tirzepatide cohort, including 1 patient with NAION.

## Discussion

In a population of patients with type 2 diabetes who had no prior diagnosis of eye diseases, this cohort study found that semaglutide or tirzepatide compared with other antidiabetic medications was associated with a differential risk of optic nerve and visual pathways, including increased risk of NAION and other optic nerve disorders, but not optic neuritis, papilledema, optic atrophy, or optic disc orders. Cumulative incidence curves began to diverge immediately after medication initiation and continued to separate thereafter. The slope of the cumulative incidence curve for the semaglutide or tirzepatide group was consistently larger than that for the comparison group, indicating the potential sustained association of semaglutide or tirzepatide with risk of NAION and other optic nerve disorders.

Our finding of increased risk of NAION is consistent with findings from cohort studies from 2024 and 2025. Hathaway et al^11^ showed in a single-center 2024 cohort study comparing semaglutide with non-GLP-1RA antidiabetic medications that semaglutide was associated with increased risk of NAION in 403 matched patients with type 2 diabetes (HR, 4.28 [95% CI, 1.62-11.29]) and 613 matched patients with obesity (HR, 7.64 [95% CI, 2.21-26.36]), with a temporal association observed within the first year. In a cohort study^13^ that was conducted in 2024 using TriNetX network, semaglutide was not associated with an increased risk of NAION in patients with type 2 diabetes compared with non-GLP-1RA antidiabetic medications but had an HR of 2.32 (95% CI, 0.60-8.97). In a 2025 multicenter cohort study,^15^ semaglutide was not associated with an increased NAION risk among individuals with type 2 diabetes compared with non-GLP-1RA antidiabetics but had an HR of 1.44 (95% CI, 0.78-2.68). In a 2024 Danish cohort study^38^ of 424 152 patients with type 2 diabetes, semaglutide was associated with an increased risk of NAION (HR, 2.19 [95% CI, 1.54-3.12]). We replicated the previous finding of an increased risk of NAION in our large cohort of 159 398 propensity score matched patients with type 2 diabetes (79 699 patients in each matched cohort). The increased risk (HR, 1.76 [95% CI, 1.01-3.07]) was significant, although the HR was smaller than in most previously reported associations. Results were consistent regardless of comparison groups (GLP-1RAs and non-GLP-1RA antidiabetic medications). In addition, we showed that the associations were sustained over the entire 2-year follow-up period. However, the CI was still wide, and future studies with larger sample sizes are needed.

In our study, we found that patients with type 2 diabetes who were prescribed semaglutide or tirzepatide had various levels of risk of optic nerve and visual pathway disorders, with increased risk of NAION and other optic nerve disorders and no associations with optic neuritis, papilledema, optic atrophy, or optic disc disorders. Each optic nerve and visual pathway disorder has distinct causes. NAION is due to the lack of blood flow to the optic nerve caused by hypertension, hypercholesterolemia, diabetes, cardiovascular and cerebrovascular disease, and obstructive sleep apnea.^34,39^ Optic neuritis is an inflammatory demyelinating disorder of the optic nerve due to multiple sclerosis, autoimmune disorders, or infections.^40^ Optic atrophy is the degeneration of the optic nerve due to ischemia, trauma, genetic conditions, glaucoma, optic neuritis, or toxic effects.^41^ Papilledema is optic disc swelling due to high intracranial pressure caused by intracerebral mass lesions, cerebral hemorrhage, head trauma, hydrocephalus, and idiopathic intracranial hypertension.^42^ Given that each optic nerve and visual pathway disorder has distinct causes, the observed differential risks associated with semaglutide and tirzepatide are expected, although future work is needed to understand the underlying mechanisms.

GLP-1 receptors are expressed in the optic nerve^43^ and as such, could be directly affected by GLP1-R agonist drugs. We performed a head-to-head comparison of semaglutide or tirzepatide with other GLP-1RAs and found that semaglutide or tirzepatide was associated with increased risk of NAION and other optic nerve disorders. For the direct comparison of semaglutide with tirzepatide, the sample size was limited and the number of patients with outcomes in both cohorts was small, preventing statistical significance testing. These results suggest that underlying mechanisms may be specific to these 2 medications not the GLP-1RA class, which may include their indirect associations with abrupt changes in metabolic parameters. While subgroup analysis based on risk factors, such as hypertension, could offer relevant insights, we could not stratify patients due to imbalanced distribution of these risk factors in the study population. For example, 76% of individuals in the study population had hypertension so the sample size of patients without hypertension was limited. Future research into mechanisms driving the increased risk will inform clinical strategies to mitigate it. Additional work to examine potential modifications by race and ethnicity and gender may be important to identify populations at increased risk. In the future, with routine clinical access to genetic analyses, it may be possible to identify individuals at increased risk for these potential complications.^44^

### Limitations

Our study has several limitations. First, retrospective observational studies using patient EHRs can have inherent limitations, including unmeasured or uncontrolled confounding and biases. We conducted rigorous target trial emulation, performed sensitivity analyses using negative control outcomes, quantified *E*-values to assess the potential impact of unmeasured confounding, and validated previous findings of the association of semaglutide or tirzepatide with increased risk of NAION.^11,15^ However, inherent limitations of retrospective EHR data analyses could not be eliminated and this was an observational study, so causal conclusions cannot be drawn from our findings. Second, our patient cohort was sourced from the TriNetX network. Validation of results in other EHR databases and analytics platforms is warranted. Third, this study focused on patients with type 2 diabetes for a 2-year follow-up. Future studies should explore longer follow-up periods and different populations. Fourth, *ICD-10*–based diagnoses for NAION and other diseases could have limitations due to overdiagnosis, misdiagnosis, and underdiagnosis. Because there is no specific *ICD-10* code for NAION, we used *ICD-10* code H47.01, “ischemic optic neuropathy” for NAION, which could have overdiagnosed NAION.^11^ Hathaway et al^11^ used this code plus manual medical record review for NAION confirmation. Cai et al^15^ used the clinical code for ischemic optic neuropathy for sensitive definition and a second confirmatory ischemic optic neuropathy diagnosis code within 90 days for a specific definition and showed that findings were largely consistent across the 2 NAION definitions. In our study, we had no access to patient-level data and could not perform manual review to confirm NAION diagnoses. We used the TriNetX analytics platform and its built-in statistical functions, which do not allow users to specify 2 outcomes within certain days. Nonetheless, our findings were consistent with previously reported findings.^11,15^ Fifth, outcomes in this study were a mixture of general and specific disease terms. This study had 2 goals: to validate previous findings about an association of semaglutide with NAION in a larger sample size and a target trial emulation framework and to examine the association of semaglutide and tirzepatide with other, related optic nerve and visual pathway disorders, which have not been studied, to our knowledge. We used *ICD-10* code ontology, which is a tree-structured organization of disease terms and their hierarchical relationships. In a hypothesis-free manner, we started with more general term, *disorders of optic nerve and visual pathways* (*ICD-10* code H46-H47, and then focused on more specific codes along the tree. We used this strategy to systematically identify where semaglutide or tirzepatide may be associated with optic nerve and visual pathways. We found that these drugs preferably had associations with optic nerves but not other visual pathways. Findings for this study could set a foundation for future hypothesis-driven approaches to focus on specific eye disorders. Sixth, EHRs in TriNetX lack data on medication adherence. Practice pattern variations among health care organizations, and patient health care use could not explicitly be controlled. However, sensitivity analyses indicated similar health care use levels between exposure and comparison groups, minimizing potential surveillance bias.

## Conclusions

This cohort study found that treatment with semaglutide or tirzepatide was associated with increased risk of optic nerve disorders, including NAION, but no other optic nerve or visual pathway disorder in patients with diabetes. However, the overall risk remained very low. Future studies are needed to replicate these findings, explore underlying mechanisms, identify individuals at increased risk for these potential complications, and examine other eye disorders.

## Notes

Supplement 1. **eAppendix.** TriNetX analytics platform **eTable 1.** Specification and emulation of pragmatic target trials **eTable 2.** Eligibility criteria and exposure definitions **eTable 3.** Outcome definitions **eTable 4.** Definitions of covariates **eTable 5.** Characteristics of semaglutide group and comparison group before and after propensity score matching **eFigure 1.** Graphical illustration of study design **eFigure 2.** Propensity score matching and density function before and after matching semaglutide or tirzepatide and other antidiabetic medication groups on listed variables in eTable 4 **eFigure 3.** Propensity score matching and density function before and after matching semaglutide or tirzepatide and other GLP-1RA groups on listed variables in eTable 4

Supplement 2.
Data Sharing Statement
