# Supplementary material for: Semaglutide or Tirzepatide and Optic Nerve and Visual Pathway Disorders in Type 2 Diabetes
Source: JAMA Netw Open. 2025 Aug 11;8(8):e2526327. doi: 10.1001/jamanetworkopen.2025.26327 (PMC12340659; doi:10.1001/jamanetworkopen.2025.26327)
Supplement: Supplement 1. — eAppendix. TriNetX analytics platform eTable 1. Specification and emulation of pragmatic target trials eTable 2. Eligibility criteria and exposure definitions eTable 3. Outcome definitions eTable 4. Definitions of covariates eTable 5. Characteristics of semaglutide group and comparison group before and after propensity score matching eFigure 1. Graphical illustration of study design eFigure 2. Propensity score matching and density function before and after matching semaglutide or tirzepatide and other antidiabetic medication groups on listed variables in eTable 4 eFigure 3. Propensity score matching and density function before and after matching semaglutide or tirzepatide and other GLP-1RA groups on listed variables in eTable 4 [file jamanetwopen-e2526327-s001.pdf]

Supplemental Online Content

Wang L, Volkow ND, Kaelber DC, Xu R. Semaglutide or tirzepatide and optic nerve and visual pathway disorders in type 2 diabetes. *JAMA Netw Open*. 2025;8(8):e2526327.
doi:10.1001/jamanetworkopen.2025.26327

eAppendix. TriNetX analytics platform

eTable 1. Specification and emulation of pragmatic target trials

eTable 2. Eligibility criteria and exposure definitions

eTable 3. Outcome definitions

eTable 4. Definitions of covariates

eTable 5. Characteristics of semaglutide group and comparison group before and after propensity score matching

eFigure 1. Graphical illustration of study design

eFigure 2. Propensity score matching and density function before and after matching semaglutide or tirzepatide and other antidiabetic medication groups on listed variables in eTable 4

eFigure 3. Propensity score matching and density function before and after matching semaglutide or tirzepatide and other GLP-1RA groups on listed variables in eTable 4

This supplemental material has been provided by the authors to give readers additional information about their work.

© 2025 Wang L et al. *JAMA Network Open*.

TriNetX Analytics Platform
The data used in this study were collected and analyzed in February 2025 within the TriNetX Analytics platform based on the "US Collaborative Network". We used the TriNetX platform to access aggregated and de-identified electronic health records (EHRs) of 118 million patients from 69 healthcare organizations (49 academic, 19 non-academic, 1 unknown) in the US across 50 states, covering diverse geographic regions, age, race/ethnic, income and insurance groups and clinical setting. Sources of patient records were distributed across the United States (30% Northeast, 16% Midwest, 36% South, 14% West, 5% Unknown).

TriNetX is a platform that de-identifies and aggregates EHRs data from contributing healthcare systems, most of which are large academic medical institutions with both inpatient and outpatient facilities at multiple locations, across all 50 states in the US. TriNetX Analytics provides web-based and secure access to patient EHR data from hospitals, primary care, and specialty treatment providers, covering diverse geographic locations, age groups, racial and ethnic groups, income levels and insurance types including various commercial insurances, governmental insurance (Medicare and Medicaid), self-pay/uninsured, worker compensation insurance, military/VA insurance among others. As a federated network, TriNetX received a waiver from Western IRB since only aggregated counts, statistical summaries of de-identified information, but no protected health information is received, and no study-specific activities are performed in retrospective analyses.

Self-reported sex (female, male), race and ethnicity data in TriNetX comes from the underlying clinical EHR systems of the contributing healthcare systems. TriNetX maps race and ethnicity data from the contributing healthcare systems to the following categories: (1) Race: Asian, American Indian or Alaskan Native, Black or African American, Native Hawaiian or Other, White, Unknown race; and (2) Ethnicity: Hispanic or Latino, Not Hispanic or Latino, Unknown Ethnicity.

TriNetX completes an intensive data preprocessing stage to minimize missing values. TriNetX maps the data to a consistent clinical data model with a consistent semantic meaning so that the data can be queried consistently regardless of the underlying data source. All covariates are either binary, categorical (which expands to a set of binary columns), or continuous but essentially guaranteed to exist. Missing sex values are represented using "Unknown Sex". The missing data for race and ethnicity are presented as "Unknown race" or "Unknown Ethnicity". For other variables including medical conditions, procedures, lab tests and socio-economic determinant health, the value is either present or absent so "missing" is not pertinent.

Supplement Table 1. Specification and emulation of pragmatic target trials: Comparing semaglutide with other antidiabetic medications for the risk of optic nerve and visual pathway disorders in patients with T2D who had no prior diagnosis of eye disorders using EHR data and analytics functions from the TriNetX Analytics Platform. Target trial specifications and emulations were similar unless otherwise stated.

Protocol	Specification of the Hypothetical Target Trial	Emulation of the Hypothetical Target Trial
Eligibility criteria	• Had medical encounters with healthcare organizations between	Same as for the target trials

© 2025 Wang L et al. *JAMA Network Open*.

	December 1, 2017 and January 31, 2023<br>• Had a diagnosis of T2D<br>• Had recent medical encounters for T2D diagnosis in the past year on or before the baseline (time of randomization).<br>• No diagnosis of any eye disorders anytime on or before the baseline.<br>• had at least one of the diseases based on the prescription guideline for semaglutide (obesity, hypertension, hypercholesterolemia, hyperlipidemia, heart diseases, chronic kidney disease, stroke or A1C ≥=8.5%) anytime on or before the baseline.<br>• No contraindication, warning, and limited use where one drug would be preferred over the other (pancreatitis, type 1 diabetes, thyroid cancer, and gastroparesis) anytime on or before the baseline.<br>• Not initiation of semaglutide/tirzepatide and the comparison antidiabetic medications at the same time at the baseline.	The baseline (the time zero or the index event) was defined as the first prescription of semaglutide/tirzepatide for the treatment cohort and the first prescription of other antidiabetic medications between December 2017 and January 2023. Eligiblity criteria was assessed based on the information on or before the baseline.
Treatment strategies	For the target trial comparing semaglutide/tirzepatide vs other antidiabetic medications<br>• Initiate use of semaglutide or tirzepatide but not initiate other antidiabetic medications at the index event (time zero)<br>• Initiate use of other antidiabetic medications (other GLP-1RAs, insulin, metformin, DPP-4i, SGLT2i, SU, or TZD) but not initiate semaglutide/tirzepatide at the index event (time zero)	Same as for the target trials. The date of medication initiation was defined as the date of a first medication prescription.

© 2025 Wang L et al. *JAMA Network Open*.

	For the target trial comparing semaglutide/tirzepatide vs other GLP-1RAs<br>• Initiate use of semaglutide or tirzepatide but not initiate other antidiabetic medications at the index event (time zero)<br>• Initiate use of other GLP-1RAs but not initiate semaglutide/tirzepatide at the index event (time zero)	
**Treatment assignment**	Individuals are randomly assigned to a treatment strategy at baseline. Individuals will be aware of the assigned treatment strategies.	Individuals are assigned to the strategy compatible with their first prescription and assumed randomization by propensity-score matching for baseline covariates.
**Outcomes**	Overall and subcategories of optic nerve and visual pathways disorders:<br>• Disorders of optic nerve and visual pathways<br>• Optic neuritis<br>• Other disorders of optic nerve and visual pathways<br>• Disorders of optic nerve<br>• NAION<br>• Other optic nerve disorders<br>• Papilledema<br>• Optic atrophy<br>• Other disorders of optic disc<br><br>Negative control outcomes as sensitivity analysis:<br>• Congenital eye diseases<br>• Any eye diseases<br>• Outpatient medical encounters<br>• Overall medical encounters.	Same as for the target trials
**Follow-up**	For each outcome, follow-up for each individual will start at 1 day following the baseline (time of the randomization) and end on day of the outcome, death, loss to follow-up, or 2 years after baseline, whichever occurs first.	Same as for the target trials.<br><br>The baseline (the time zero or the index event) was defined as the first prescription of semaglutide/tirzepatide for the treatment cohort and the first prescription of other antidiabetic medications between December

© 2025 Wang L et al. *JAMA Network Open*.

		2017 and January 2023. Eligiblity criteria was assessed based on the information on or before the baseline.
**Casual contrast of interest**	Intention-to-treat	Observational analog to intention-to-treat
**Statistical analysis**	• Kaplan-Meier estimator to obtain cumulative incidences for each treatment strategy within 2 year of follow-up.<br>• Cox proportional hazards analyses to compare rates of time-to-events on daily basis during follow-up time since the baseline.	Same as for the target trial except observational analogs of intention-to-treat analyses required matching for confounding variables by propensity-score matching.<br><br>Models are adjusted for confounders at baseline

DPP-4i denotes dipeptidyl-peptidase-4 inhibitors; SGLT2i sodium-glucose cotransporter-2 inhibitors, SU for sulfonylureas, TZD for thiazolidinediones. Other GLP-1RAs include albiglutide, dulaglutide, exenatide, liraglutide, and lixisenatide.

Supplement Table 2. Eligibility criteria and exposure definitions.

**Eligibility criteria**		
**Variable**	**Values**	**Name and Codes**
Diagnosis of T2D	Binary: present/absent	Type 2 diabetes mellitus (ICD-10 code: E11)
No prior diagnosis of eye disorders	Binary: present/absent	Diseases of the eye and adnexa (ICD-10 code: H00-H59)
Had at least one of the diseases based on the prescription guideline for semaglutide (obesity, hypertension, hypercholesterolemia, hyperlipidemia, heart diseases, stroke, chronic kidney disease, A1C ≥8.5%).	Binary: present/absent	• Hypertension (ICD-10: I10-I1A)<br>• Hypercholesterolemia (ICD-10 E78.0)<br>• Hyperlipidemia (ICD-10: E78.2, E78.4, E78.5)<br>• Heart diseases (ICD-10: I20-I25, I30-I5A)<br>• Stroke (ICD-10: I63, I60-I69)<br>• Obesity (E66.0, E66.2, E66.8, E66.9, Z68.3, Z68.4)<br>• Chronic kidney disease (ICD-10: N18)<br>• Hemoglobin A1c/Hemoglobin.total in

© 2025 Wang L et al. *JAMA Network Open*.

		Blood (at least 8.50 %) (TNX:9037)
No contraindication, warning, and limited use where one drug would be preferred over the other (pancreatitis, type 1 diabetes, thyroid cancer, and gastroparesis)	Binary: present/absent	• Pancreatitis (ICD-10: K85, K86.0, K86.1)<br>• Type 1 diabetes (ICD-10: E10)<br>• Gastroparesis (ICD-10: K31.84)<br>• Thyroid cancer (ICD-10: C73, Z85.850, E31.2)
No initiation of semaglutide/tirzepatide with the other antidiabetic medications at the same time at time zero		•
Exposure definitions		
Initiate use of semaglutide or tirzepatide at baseline	Binary: present/absent	• Semaglutide (RxNorm code: 1991302)<br>• Tirzepatide (RxNorm code: 2601723)
Initiate use of other antidiabetic medications at baseline	Binary: present/absent	• Albiglutide: RxNorm code: 1534763<br>• Exenatide: RxNorm code: 60548<br>• Dulaglutide: RxNorm code:1551291<br>• Liraglutide: RxNorm code: 475968<br>• Lixisenatide: RxNorm code: 1440051<br>• Insulins (ATC code: A10A)<br>• Metformin (ATC code: A10BA)<br>• Dipeptidyl peptidase 4 (DPP-4) inhibitors (ATC code: A10BH)<br>• Sodium-glucose co-transporter 2 (SGLT2) inhibitors (ATC code: A10BK)<br>• Sulfonylureas (ATC code: A10BB)<br>• Thiazolidinediones (ATC code: A10BF)
Initiate use of other GLP-1RA at baseline	Binary: present/absent	• Albiglutide: RxNorm code: 1534763

© 2025 Wang L et al. *JAMA Network Open*.

		• Exenatide: RxNorm code: 60548<br>• Dulaglutide: RxNorm code:1551291<br>• Liraglutide: RxNorm code: 475968<br>• Lixisenatide: RxNorm code: 1440051
Initiate use of semaglutide at baseline	Binary: present/absent	Semaglutide (RxNorm code: 1991302)
Initiate use of tirzepatide at baseline	Binary: present/absent	Tirzepatide (RxNorm code: 2601723)

ICD-10: International Classification of Diseases System, version 10
RxNorm: medical prescription normalized Medical prescription
ATC: Anatomical Therapeutic Chemical (ATC) classification system
CPT: Current Procedural Terminology

Supplement Table 3. Outcome definitions.

**Eligibility criteria**		
**Variable**	**Values**	**Name and Codes**
**Primary outcomes**		
Disorders of optic nerve and visual pathways	Binary: present/absent	Disorders of optic nerve and visual pathways (ICD-10 code: H46-H47)
Optic neuritis	Binary: present/absent	Optic neuritis (ICD-10 code: H46)
Other disorders of optic [2nd] nerve and visual pathways	Binary: present/absent	Other disorders of optic [2nd] nerve and visual pathways (ICD-10 code: H47)
Disorders of optic nerve	Binary: present/absent	Disorders of optic nerve, not elsewhere classified (ICD-10 code: H47.0)
NAION	Binary: present/absent	Ischemic optic neuropathy (ICD-10 code: H47.01)
Disorders of optic nerve	Binary: present/absent	• Hemorrhage in optic nerve sheath (ICD-10 code: H47.02)<br>• Optic nerve hypoplasia (ICD-10 code: H47.03)<br>• Other disorders of optic nerve, not elsewhere

© 2025 Wang L et al. *JAMA Network Open*.

		classified (ICD-10 code: H47.09)
Papilledema	Binary: present/absent	Papilledema (ICD-10 code: H47.1)
Optic atrophy	Binary: present/absent	Optic atrophy (ICD-10 code: H47.2)
Other disorders of optic disc	Binary: present/absent	Other disorders of optic disc (ICD-10 code: H47.3)
**Negative Control outcomes**		
Congenital eye disorders	Binary: present/absent	• Congenital malformations of eyelid, lacrimal apparatus and orbit (ICD10 code: Q10)<br>• Congenital lens malformations (ICD10 code: Q12)<br>• Congenital malformations of anterior segment of eye (ICD10 code: Q13)<br>• Congenital malformations of posterior segment of eye (ICD10 code: Q14)<br>• Other congenital malformations of eye (ICD10 code: Q15).
All eye disorders	Binary: present/absent	Diseases of the eye and adnexa (ICD10 code: H00-H59).
Outpatient medical visit	Binary: present/absent	Visit: Ambulatory (HL7V3.0:VisitType:AMB)
Outpatient medical visit	Binary: present/absent	Visit (TNX:Visit)

ICD-10: International Classification of Diseases System, version 10
RxNorm: medical prescription normalized Medical prescription
ATC: Anatomical Therapeutic Chemical (ATC) classification system
HL7V3.0: Health Level Seven (HL7) Vocabulary, Version 3.0

Supplement Table 4: Definitions of covariates.

Variable	Value	Code	Coding terminology
Age at Index	continuous	AI	Demographics
Female	Binary: present/absent	F	Demographics
Male	Binary: present/absent	M	Demographics

© 2025 Wang L et al. *JAMA Network Open*.

Black or African American	Binary: present/absent	2054-5	Demographics
White	Binary: present/absent	2106-3	Demographics
Unknown Race	Binary: present/absent	UNK	Demographics
Unknown Gender	Binary: present/absent	UN	Demographics
Not Hispanic or Latino	Binary: present/absent	2186-5	Demographics
Hispanic or Latino	Binary: present/absent	2135-2	Demographics
Asian	Binary: present/absent	2028-9	Demographics
Persons with potential health hazards related to socioeconomic and psychosocial circumstances	Binary: present/absent	Z55-Z65	ICD-10
Problems related to lifestyle	Binary: present/absent	Z72	ICD-10
Type 2 diabetes mellitus with neurological complications	Binary: present/absent	E11.4	ICD-10
Type 2 diabetes mellitus with circulatory complications	Binary: present/absent	E11.5	ICD-10
Type 2 diabetes mellitus with kidney complications	Binary: present/absent	E11.2	ICD-10
Type 2 diabetes mellitus with ophthalmic complications	Binary: present/absent	E11.3	ICD-10
Type 2 diabetes mellitus with other specified complications	Binary: present/absent	E11.6	ICD-10
Type 2 diabetes mellitus with unspecified complications	Binary: present/absent	E11.8	ICD-10
Overweight and obesity	Binary: present/absent	E66	ICD-10
Metabolic disorders	Binary: present/absent	E70-E88	ICD-10
Hypertension	Binary: present/absent	I10-I1A	ICD-10
Hypotension	Binary: present/absent	I95	ICD-10
Disorders of lipoprotein metabolism and other lipidemias	Binary: present/absent	E78	ICD-10
Ischemic heart diseases	Binary: present/absent	I20-I25	ICD-10
Other forms of heart disease	Binary: present/absent	I30-I5A	ICD-10
Cerebrovascular diseases	Binary: present/absent	I60-I69	ICD-10
Kidney diseases	Binary: present/absent	N17-N19	ICD-10

© 2025 Wang L et al. *JAMA Network Open*.

Atherosclerosis	Binary: present/absent	I70	ICD-10
Sleep apnea	Binary: present/absent	G47.3	ICD-10
Migraine	Binary: present/absent	G43	ICD-10
Coagulation defects, purpura and other hemorrhagic conditions	Binary: present/absent	D65-D69	ICD-10
Alcohol use disorder	Binary: present/absent	F10	ICD-10
Tobacco use disorder	Binary: present/absent	F17	ICD-10
Antihypertensives	Binary: present/absent	C02	ATC
Antihypertensives, others	Binary: present/absent	CV490	VA
Antiarrhythmics	Binary: present/absent	CV300	VA
Amphetamines	Binary: present/absent	CN801	VA
aspirin	Binary: present/absent	1191	RxNorm
sildenafil	Binary: present/absent	136411	RxNorm
tadalafil	Binary: present/absent	358263	RxNorm
amiodarone	Binary: present/absent	703	RxNorm
Interferons	Binary: present/absent	L01AB	ATC
Insulins	Binary: present/absent	A10A	ATC
Metformin	Binary: present/absent	A10BA	ATC
Dipeptidyl peptidase 4 (DPP-4) inhibitors	Binary: present/absent	A10BH	ATC
Sodium-glucose co-transporter 2 (SGLT2) inhibitors	Binary: present/absent	A10BK	ATC
Sulfonylureas	Binary: present/absent	A10BB	ATC
Thiazolidinediones	Binary: present/absent	A10BF	ATC
GLP-1RAs	Binary: present/absent	A10BJ	ATC
Outpatient medical visits	Binary: present/absent	AMB	HL7V3.0
Inpatient medical visits	Binary: present/absent	IMP	HL7V3.0
Emergency visits	Binary: present/absent	EMER	HL7V3.0

ICD-10: International Classification of Diseases System, version 10
RxNorm: medical prescription normalized Medical prescription
ATC: Anatomical Therapeutic Chemical (ATC) classification system
VA: Veterans Affairs Drug Classification system
CPT: Current Procedural Terminology
HL7V3.0: Health Level Seven (HL7) Vocabulary, Version 3.0

Supplement Table 5: Characteristics of the semaglutide group and the comparison group before and after propensity-score matching.

	**Before Propensity-Score Matching**			**After Propensity-Score Matching**		
	**Semaglutide/ tirzepatide**	**Other GLP-1RAs**	**SMD**	**Semaglutide/ tirzepatide**	**Other GLP-1RAs**	**SMD**

© 2025 Wang L et al. *JAMA Network Open*.

**Total number**	82 972	124 908		71 459	71 459	
**Age at index event, mean ± SD, y**	56.6 ± 12.4	58.4 ± 13.3	0.14*	57.0 ± 12.5	57.0 ± 13.3	0.006
**Sex, No. (%)**						
Female	42 994 (51.8)	60 493 (48.4)	0.07	36 442 (51.0)	36 459 (51.0)	<.001
Male	35 017 (42.2)	59 555 (47.7)	0.11*	31 212 (43.7)	31 211 (43.7)	<.001
Unknown	4 958 (6.0)	4 860 (3.9)	0.10*	3 805 (5.3)	3 789 (5.3)	0.001
**Ethnicity, No. (%)**						
Hispanic/Latinx	5 849 (7.0)	13 072 (10.5)	0.12*	5 497 (7.7)	5 579 (7.8)	0.004
Not Hispanic/Latinx	58 747 (70.8)	80 895 (64.8)	0.13*	49 811 (69.7)	49 498 (69.3)	0.01
Unknown	18 375 (22.1)	30 941 (24.8)	0.06	16 151 (22.6)	16 382 (22.9)	0.008
**Race, No. (%)**						
Asian	3 983 (4.7)	3 791 (3.0)	0.09	2 821 (3.9)	2 755 (3.9)	0.005
Black	12 904 (15.6)	21 861 (17.5)	0.05	11 581 (16.2)	11 699 (16.4)	0.004
White	52 963 (63.8)	77 537 (62.1)	0.04	45 681 (63.9)	45 599 (63.8)	0.002
Unknown	8 563 (10.3)	14 063 (11.3)	0.03	7 312 (10.2)	7 387 (10.3)	0.003
**Adverse socioeconomic determinants of health, No. (%)**	2 460 (3.0)	3 244 (2.5)	0.02	2 039 (2.9)	2 043 (2.9)	<.001
**Problems related to lifestyle, No. (%)**	5 941 (7.2)	7 901 (6.3)	0.03	4 953 (6.9)	5 131 (7.2)	0.01
**T2DM complications, No. (%)**						
Type 2 diabetes mellitus with kidney complications	12 497 (15.1)	20 068 (16.1)	0.03	10 829 (15.2)	11 087 (15.5)	0.01
Type 2 diabetes mellitus with ophthalmic complications	2 993 (3.6)	5 394 (4.3)	0.04	2 713 (3.8)	2 801 (3.9)	0.006
Type 2 diabetes mellitus with neurological complications	13 277 (16.0)	23 305 (18.7)	0.07	12 028 (15.8)	12 389 (16.2)	0.01
Type 2 diabetes mellitus with circulatory complications	6 337 (7.6)	8 950 (7.2)	0.02	5 344 (7.5)	5 377 (7.5)	0.002
Type 2 diabetes mellitus with other specified complications	40 942 (49.3)	59 826 (47.9)	0.03	35 010 (49.0)	35 803 (50.1)	0.02
Type 2 diabetes mellitus with unspecified complications	9 368 (11.3)	14 277 (11.4)	0.004	8 125 (11.4)	8 288 (11.6)	0.007
**Pre-existing medical conditions, No. (%)**						
Overweight and obesity	48 286 (58.2)	54 354 (43.5)	0.30*	38 862 (54.4)	39 063 (54.7)	0.006

© 2025 Wang L et al. *JAMA Network Open*.

Metabolic disorders	65 428 (78.9)	87 953 (70.4)	0.20*	55 144 (77.2)	56 034 (78.4)	0.03
Hypertension	62 888 (75.8)	88 424 (70.8)	0.11*	53 595 (75.0)	54 543 (76.3)	0.03
Hypotension	3 444 (4.2)	5 745 (4.6)	0.02	3 066 (4.3)	3 075 (4.3)	0.001
Disorders of lipoprotein metabolism and other lipidemias	62 182 (74.9)	82 308 (65.9)	0.20*	52 269 (73.1)	53 131 (74.4)	0.03
Ischemic heart diseases	15 120 (18.2)	25 829 (20.7)	0.06	13 578 (19.0)	13 781 (19.3)	0.007
Other forms of heart disease	21 779 (26.2)	32 214 (25.8)	0.01	18 672 (26.1)	18 843 (26.4)	0.005
Cerebrovascular diseases	5 583 (6.7)	10 285 (8.2)	0.06	5 072 (7.1)	5 155 (7.2)	0.005
Kidney diseases	13 680 (16.5)	24 161 (19.3)	0.08	12 280 (17.2)	12 630 (17.7)	0.01
Atherosclerosis	3 491 (4.2)	5 683 (4.5)	0.02	3 105 (4.3)	3 190 (4.5)	0.006
Sleep apnea	24 168 (29.1)	26 283 (21.0)	0.44*	19 032 (28.4)	19 155 (29.0)	0.004
Migraine	5 853 (7.1)	5 480 (4.4)	0.12*	4 251 (5.9)	4 304 (6.0)	0.003
Coagulation defects, purpura and other hemorrhagic conditions	3 934 (4.7)	5 461 (4.4)	0.02	3 341(4.7)	3 418 (4.8)	0.005
Alcohol use disorder	1 728 (2.1)	2 948 (2.4)	0.02	1 543 (2.2)	1 504 (2.1)	0.004
Tobacco use disorder	10 215 (12.3)	16 037 (12.8)	0.02	8 979 (12.6)	9 142 (12.8)	0.007
**Prior medications (%)**						
Antihypertensives	12 413 (15.0)	17 205 (13.8)	0.03	10 375 (14.5)	10 505 (14.7)	0.005
Antihypertensives, others	17 331 (20.9)	22 381 (17.9)	0.08	14 287 (20.0)	14 427 (20.2)	0.005
Antiarrhythmics	37 024 (44.6)	43 004 (34.4)	0.21*	29 504 (41.3)	29 601 (41.4)	0.003
Amphetamines	2 441 (2.9)	1 902 (1.5)	0.10*	1 618 (2.3)	1 621 (2.3)	<.001
aspirin	25 052 (30.2)	39 107 (31.3)	0.02	21 945 (30.7)	22 348 (31.3)	0.01
sildenafil	5 157 (6.2)	5 761 (4.6)	0.07	4 162 (5.8)	4 178 (5.8)	0.001
tadalafil	3 204 (3.9)	17 719 (1.2)	0.17*	2 483 (3.5)	2 492 (3.5)	0.001
amiodarone	1 685 (2.0)	2 641 (2.1)	0.006	1 513 (2.1)	1 508 (2.1)	0.001
Interferons	77 (0.1)	142 (0.1)	0.007	65 (0.1)	81 (0.1)	0.07
Insulin	34 773 (41.9)	60 046 (48.1)	0.12*	31 309 (43.8)	32 146 (45.0)	0.02
Metformin	57 029 (69.0)	72 506 (58.0)	0.23*	47 517 (66.5)	48 376 (67.7)	0.03
Dipeptidyl peptidase 4 (DPP-4) inhibitors	15 991 (19.3)	22 989 (18.4)	0.02	13 732 (19.2)	14 064 (19.7)	0.01
Sodium-glucose co-transporter 2 (SGLT2) inhibitors	21 453 (25.9)	20 545 (16.4)	0.23*	16 222 (22.7)	16 242 (22.7)	0.001
Sulfonylureas	23 155 (27.9)	36 233 (29.0)	0.02	20 400 (28.5)	21 117 (29.6)	0.02
Other GLP-1RAs	20 960 (25.3)	17 013 (13.6)	0.30*	14 589 (20.4)	14 566 (20.4)	0.001
**Medical encounters, No. (%)**						
Outpatient medical visits	69 369 (83.6)	94 967 (76.0)	0.19*	59 247 (82.9)	59 967 (83.9)	0.03
Inpatient medical	22 515 (27.1)	34 529 (27.6)	0.01	19 618 (27.5)	19 810 (27.7)	0.006
Emergency visits	31 489 (38.0)	40 096 (32.1)	0.12*	25 718 (36.0)	25 850 (36.2)	0.004

© 2025 Wang L et al. *JAMA Network Open*.

Shown are groups before and after propensity-score matching for the listed variables. The status of variables was based on the presence of related clinical codes anytime up to 1 day before the index event (first prescription of semaglutide or insulin during 12/2017-1/2023). SMD – standardized mean differences. *SMD greater than 0.1, a threshold indicating cohort imbalance. SD – standard deviation. Adverse socioeconomic determinants of health included housing and economic circumstances, upbringing, education, physical environment, and social environment. Problems with lifestyle included tobacco use, lack of physical exercise, inappropriate diet and eating habits, and others.

Supplement Figure 1. Graphical illustration of the study design.

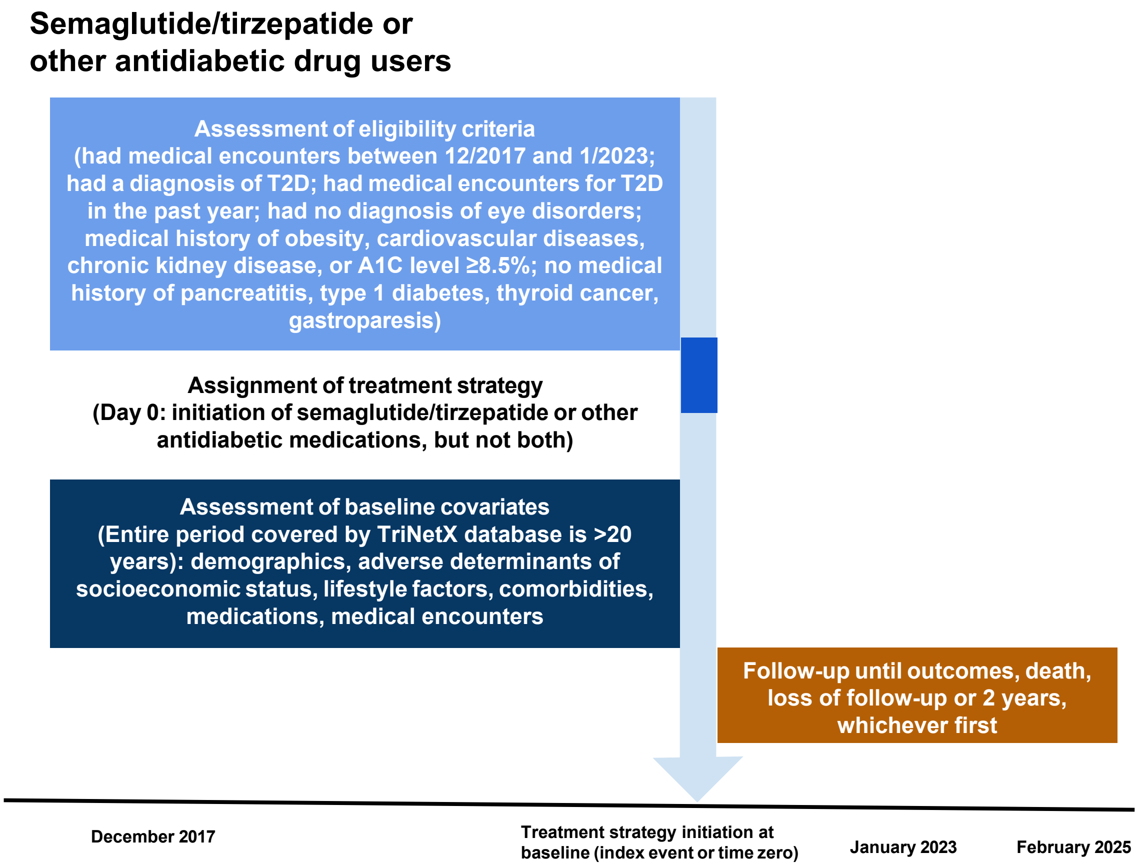

See Supplement Table 2 for definitions of eligibility criteria, exposure, covariates, and outcomes. Follow-up for each individual started 1 day following the treatment assignment and ended on the day of outcome, death, loss to follow-up, or 2 years after baseline, whichever occurred first.

Supplement Figure 2. Propensity score matching and density function – before and after matching the semaglutide/tirzepatide and other antidiabetic medication groups on the listed variables in Supplement Table 4.

© 2025 Wang L et al. *JAMA Network Open*.

**Cohort 1 and cohort 2 patient count before and after propensity score matching**		
Cohort	Patient count before matching	Patient count after matching
1 – semaglutide/tirzepatide	82,972	79,699
2 – other antidiabetic medications	1,428,665	79,699
**Propensity score density function - Before and after matching (cohort 1 - purple, cohort 2 - green)**		

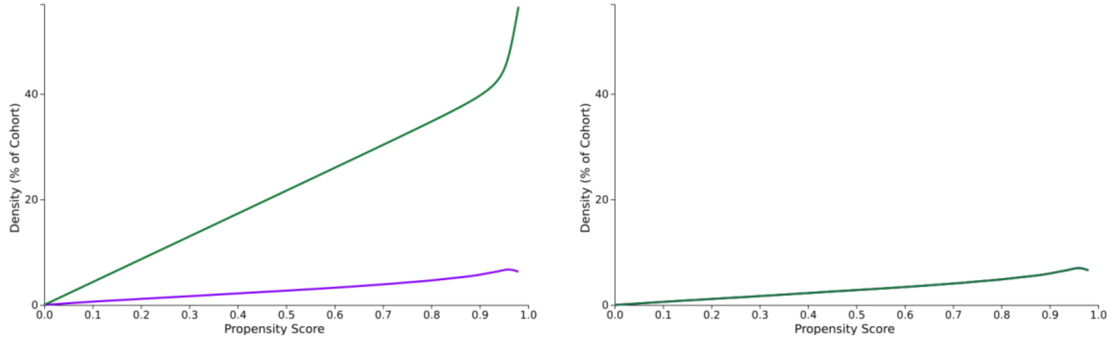

Supplement Figure 3. Propensity score matching and density function – before and after matching the semaglutide/tirzepatide and other GLP-1RA groups on the listed variables in Supplement Table 4.

**Cohort 1 and cohort 2 patient count before and after propensity score matching**		
Cohort	Patient count before matching	Patient count after matching
1 – semaglutide/tirzepatide	82,972	71,459
2 – other GLP-1RAs	124,908	71,459
**Propensity score density function - Before and after matching (cohort 1 - purple, cohort 2 - green)**		

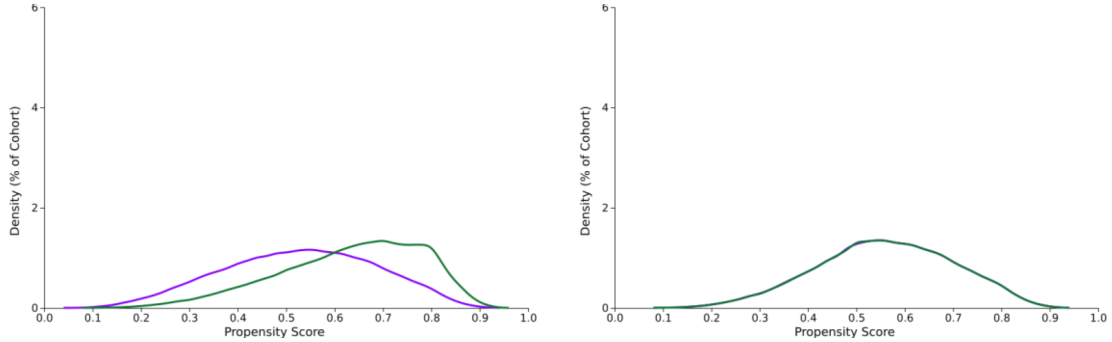

© 2025 Wang L et al. *JAMA Network Open*.
