# Supplementary material for: Semaglutide or Tirzepatide and Optic Nerve and Visual Pathway Disorders in Type 2 Diabetes
Source: JAMA Netw Open. 2025 Aug 11;8(8):e2526327. doi: 10.1001/jamanetworkopen.2025.26327 (PMC12340659; doi:10.1001/jamanetworkopen.2025.26327)
Supplement: Supplement 2. — Data Sharing Statement [file jamanetwopen-e2526327-s002.pdf]

Data Sharing Statement

Wang. Semaglutide or Tirzepatide and Optic Nerve and Visual Pathway Disorders in Type 2 Diabetes. *JAMA Netw Open*. Published online August11, 2025. doi:10.1001/jamanetworkopen.2025.26327

Data
Data available: No

Additional Information
Explanation for why data not available: This study used population-level aggregate and HIPAA de-identified data collected by the TriNetX platform ("US Collaborative Network") and available from TriNetX, LLC (https://trinetx.com/), but third-party restrictions apply to the availability of these data. The data were used under license for this study with restrictions that do not allow for the data to be redistributed or made publicly available. To gain access to the data, a request can be made to TriNetX (join@trinetx.com), but costs may be incurred, and a data-sharing agreement may be necessary. Data specific to this study including diagnosis codes and cohort characteristics in aggregated format are included in the manuscript as tables, figures, and supplementary files. Data through the TriNetX platform is queried in real-time with results being returned typically in seconds to minutes. Data from the underlying electronic health records of participating healthcare organizations is refreshed in the TriNetX platform from daily to every couple of months depending on the healthcare organization.
